# Adolescent Light Cigarette Smoking Patterns and Adult Cigarette Smoking

**DOI:** 10.1155/2016/9587340

**Published:** 2016

**Authors:** R. ConstanceWiener, Alcinda K. Trickett Shockey, Susan K. Morgan

**Affiliations:** 1 Dental Practice and Rural Health, School of Dentistry, 104A Health Sciences Center Addition, P.O. Box 9448, Morgantown, WV 26506, USA; 2 Department of Periodontics, School of Dentistry, Division of Dental Hygiene, West Virginia University, Robert C Byrd Health Sciences Center North, Room 1192A, Morgantown, WV 26506, USA; 3 Department of Periodontics, School of Dentistry, West Virginia University, Robert C Byrd Health Sciences Center North, P.O. Box 9490, Room 1070, Morgantown, WV 26506, USA

## Abstract

**Purpose:**

Light cigarette smoking has had limited research. The purpose of this study was to examine the relationship between light smoking in adolescence with smoking in adulthood.

**Methods:**

National Longitudinal Study of Adolescent Health data, Waves I and IV, were analyzed. Previous month adolescent smoking of 1–5 cigarettes/day (cpd) (light smoking); 6–16 cpd (average smoking); 17 or more cpd (heavy smoking); and nonsmoking were compared with the outcome of adult smoking.

**Results:**

At baseline, 15.9% of adolescents were light smokers, 6.8% were average smokers, and 3.6% were heavy smokers. The smoking patterns were significantly related to adult smoking. In logistic regression analyses, adolescent light smokers had an adjusted odds ratio (AOR) of 2.45 (95% CI: 2.00, 3.00) of adult smoking; adolescent average or heavy smokers had AOR of 5.57 (95% CI: 4.17, 7.43) and 5.23 (95% CI: 3.29, 8.31), respectively.

**Conclusion:**

Individuals who initiate light cigarette smoking during adolescence are more likely to smoke as young adults.

**Practical Implications:**

When screening for tobacco use by adolescents, there is a need to verify that the adolescents understand that light smoking constitutes smoking. There is a need for healthcare providers to initiate interventions for adolescent light smoking.

## 1. Introduction

In 2000–2004, tobacco was related to 443,000 deaths, 5.1 million years of potential life lost, and $96 billion in healthcare expenditures in the US [1]. Tobacco use trends have changed. Smoking prevalence has steadily declined over the previous decades [1, 2]. From 1991 to 2002, the number of people smoking 15 or more cigarettes per day (cpd) has fallen in the age groups of 18–29, 30–44, and 45–64 years [3]. However, other smoking patterns are emerging and are referred to with various descriptors such as “minimal smoking,” “light smoking,” “low-rate smoking,” “intermittent smoking,” and “chipping” [4, 5]. The terminology is problematic in that the definitions may vary from study to study. For example, some researchers have defined light smoking as smoking 1–4 cpd, while others have defined light smoking as smoking less than a pack of cpd (1–20 cpd) [3, 4, 6]. In the Clinical Practice Guideline on Treating Tobacco Use and Dependence, published by the U.S. Department of Health and Human Services, light smoking is defined as smoking fewer than 10 cpd [7]. The public often has an inaccurate perception that light smoking has no risk [5] and may not even consider light smoking as actually smoking. In a recent study, Towns and her colleagues provided evidence of the addictive nature of light smoking in adolescents [2].

Tobacco education is an important factor in nearly every school system in the US and 91.7% of US adolescents responded that they had received tobacco education [8]. However, researchers of a study of adolescents’ perceptions of light smoking indicated that only 64.3% reported that they perceived light smoking to be harmful [9]. Light smoking is associated with cardiovascular disease, increased risk of cancer [4, 10], respiratory tract infections, cataracts, risk of ectopic pregnancy and placenta previa, and poor bone mineral density [4]. Each day 3,900 children try their first cigarette; more than 950 children become regular smokers; and approximately half will eventually die from the consequences of smoking [11]. Researchers report 82% of children ages 11 to 19 years who smoked were interested in quitting; however, only 4% were annually successful [7].

Early addiction is a substantial problem in adolescence. The brains of adolescents, as compared with the brains of adults, are more susceptible to nicotine and psychoactive substance dependency due to the developmental state of the brain during adolescence. The maturation of the prefrontal cortex (PFC) of the brain occurs during adolescence and young adulthood [12]. The PFC is responsible for executive functions such as cognitive self-control of behavior and attention [12]. Nicotine exposure during PFC maturation may negatively impact normal maturation, cognitive ability, attention, mental health, and personality [12]. Additionally, there is greater vulnerability to nicotine’s effects in the brains of adolescents than the brains of adults [12].

Adolescents have greater nicotine-induced pleasurable effects from smoking than adults [13, 14]. This may result in rapid continued use [13, 14], nicotine dependence, and an increased impulse to smoke even if they are motivated to not smoke [15]. As with other addictive behaviors during adolescence, smoking has effects on cognition, neurobiology, drug-related behavior, and affect which are different from effects which occur with individuals who initiate smoking in adulthood [15].

Although there is considerable literature concerning the association of heavy smoking in adolescence as a risk factor for smoking in adulthood, there is a lack of research concerning the relationship between adolescent light smoking and smoking in adulthood, even though adolescents self-report dependence at light levels of smoking [12].

The purpose of this study was to examine the relationship between the light smoking behavioral pattern in adolescence with smoking in young adulthood.

The rationale for this study is that adolescents may misjudge their ability to quit if they consider themselves to be nonsmokers when they are actually light smokers. Additionally, light smoking in adolescence may be highly associated with smoking in young adulthood. The research hypothesis is that light smoking is a risk factor for smoking into young adulthood.

The theoretical model framework used in this study was the Lydon et al. 2014 Smoking-Specific Neurobiological Model in which previous neurobiological models have been extended to include smoking specific psychosocial factors, contextual factors, incentive, motivation, and cognitive control [15]. In the model, adolescence is a vulnerable period for risky behavior as there is increased approach motivation (exploratory behavior to novel stimuli) which is difficult to control due to the stage of brain development, lack of desire to modulate the motivation, presence of peers, emotional arousal, beliefs about cigarettes, and increased hedonic response to reward consumption [15].

## 2. Method

This study was acknowledged by the West Virginia University Institutional Review Board (protocol 1507767430). The data source was the National Longitudinal Study of Adolescent Health, 1994–2008, Waves I and IV. The National Longitudinal Study of Adolescent Health was a nationally representative study of US adolescents in grades 7–12 in 1994–5 (Wave I of the study), with 3 follow-ups. For Wave I, the researchers used a stratified cluster design to select students from 80 high schools and 52 additional feeder schools who attended school on the day that the researchers provided the questionnaire. There were 6,504 adolescents in Wave I and 5,114 adolescents in Wave IV.

For Wave IV, the researchers interviewed the young adults to obtain sociodemographic data and information regarding marriage/relationships, education, labor, and health issues. The details of the study are available at the National Longitudinal Study of Adolescent Health website (http://www.icpsr.umich.edu/icpsrweb/ICPSR/studies/21600/datasets). Waves I (1994–5) and IV (2008–9) were used in this study.

### 2.1. Sample

The inclusion criterion for the sample used in this study was complete data concerning smoking in Wave I and Wave IV. In Wave I, there were 6,472 participants who had complete data on smoking. There were 5,077 participants who had complete data on smoking in both Wave I and Wave IV and were included in this study. The lost-to-follow-up participants (*N* = 1,395) were similar to the participants who were included: there were 75.8% who did not report smoking in the previous month, 14.6% who smoked 1–5 cpd, 6.6% who smoked 6–16 cpd, and 3.1% who smoked 17 or more cpd on the days in which they smoked during the previous month.

### 2.2. Key Dependent Variable

The key dependent variable was adult smoking in Wave IV of the National Longitudinal Study of Adolescent Health. Adult smoking was defined as smoking at least one cigarette during the past 30 days. Participants were adolescent students from 80 high schools and 52 additional feeder schools, ages 11 years and above in Wave I, and ages 24 years and above in Wave IV.

### 2.3. Key Independent Variable

The key independent variable was the smoking behavioral pattern in adolescence (nonsmoking, light smoking, average smoking, and heavy smoking) based upon the number of cigarettes smoked in the previous month on the days in which the adolescent chose to smoke (i.e., not every day of the month). A nonsmoking behavioral pattern was defined as not having smoked. A light smoking behavioral pattern was defined as having smoked 1–5 cpd. This definition was based upon the definition utilized in previous research [16]. An average smoking behavioral pattern was defined as smoking 6–16 cpd. A heavy smoking behavioral pattern was defined as smoking 17 or more cpd [16].

### 2.4. Other Important Variables

Other potential confounding variables or covariates were determined by factors used in the Lydon et al. Smoking-Specific Neurobiological Theoretical Model [15] and variables which were significant in previous research [17]. The variables included (1) the participants’ three close friends’ smoking habits (no close friends smoke, 1 close friend smokes, 2 close friends smoke, and 3 close friends smoke); (2) sex (male, female); (3) race/ethnicity (non-Hispanic white, non-Hispanic black, Hispanic, and others); (4) body mass index (normal (less than 25), overweight (25 to less than 30), obese (30 and above)); (5) the frequency of dental visits (within the year, within 1–2 years, over 2 years, and never); (6) exercise during the past week (none, 1–2 times, 3–4 times, and 5 times or more); (7) adequate sleep (a yes or no self-reported response to the questions, “Do you get enough sleep?”); and (8) depression symptoms (yes, no).

National Longitudinal Study of Adolescent Health study designers used a modified version of the Center for Epidemiologic Studies Depression Scale (CES-D) to determine the presence of symptoms of adolescent depression [18]. The statements in the modified version of the scale were presented in the second person form (“you were, you felt,” etc.) rather than in the first person form (“I was, I felt,” etc.) which appeared in the Radloff version.

Several phrases were also modified. The phrase “you were too tired to do things” was used in the modified version rather than the phrase “everything I did was an effort” which was used in the Radloff version. The phrase “it was hard to get started doing things” was used in the modified version rather than the phrase “I could not get going.” The phrase “trouble falling asleep or staying asleep” was used in the modified version rather than the phrase “my sleep was restless.” The phrase “frequent crying” was used in the modified version rather than the phrase “I had crying spells,” which was used in the Radloff version.

Researchers in other studies have indicated that adolescents score higher on the CES-D than adults [19–22]. The cut point for depression symptoms in this study was 24, as used by Gotlib et al. [20] and Rushton et al. [19].

### 2.5. Statistical Analysis

Data were analyzed with SAS version 9.3 (SAS Institute Inc., Cary, NC, USA). Eligibility for inclusion in the sample for this study was restricted to participants with sample weights and adult smoking data. Sample weights for Wave IV (GSWGT4 2) and cluster weights were included in the analyses; a strata variable was not available and the researchers of the National Longitudinal Study of Adolescent Health source data, in their report on correcting for design effects, indicated that not using a strata variable only minimally affected the standard errors [8]. The 0.05 level of significance was used in the study.

## 3. Results

The sample size of the study was 5,077 participants. Details of the sample characteristics are in Table 1. In summary, there were 1,187 (25.1%) participants ages 11–14 years, 1,780 (34.7%) participants ages, 15–1 6 years, and 2,107 (40.2%) participants 17 years and above at the beginning of the National Longitudinal Study of Adolescents to Adult Health. The sample was 50.4% male, 70.1% non-Hispanic white, and 76.5% normal weight. There were 74.1% of adolescents who reported enough sleep and 68.3% who reported having a dental visit within the previous year. One-fourth (25.2%) reported exercising at least 5 times a week, and over one half (54.3%) reported having nonsmokers as their three close friends. There were 770 (14.8%) participants who had depressive symptoms.

There were 3,817 (73.7%) adolescents with a nonsmoking behavioral pattern; 787 (15.9%) adolescents with a light smoking behavioral pattern; 320 (6.8%) adolescents with an average smoking behavioral pattern; and 153 (3.6%) adolescents with a heavy smoking behavioral pattern. There were 3,248 (61.7%) young adults who did not smoke in the 2008–2009 follow-up.

Bivariate relationships with adult smoking patterns are presented in Table 2. Statistically significant relationships with adult smoking exist for male sex, non-Hispanic white race/ethnicity, body mass index, having close friends who smoke, depression symptoms, and smoking during adolescence. Adequate sleep, exercise behavior, and dental visit failed to reach significance.

The significant associations were used in building the logistic regression model, and sleep, exercise behavior, and dental visit were excluded.

The results of the logistic regressions are presented in Table 3. In the unadjusted analysis, adolescents with a light smoking behavioral pattern had an odds ratio (OR) of 3.03 (95% CI: 2.50, 3.67) of becoming young adults who smoked as compared with adolescents with a nonsmoking behavioral pattern. The unadjusted OR for the average smoking and heavy smoking behavioral patterns were 7.72 (95% CI: 5.82, 10.22) and 7.88 (95% CI: 5.09, 11.89), respectively. The association was attenuated in the adjusted odds ratios (AOR) with the additional factors of adolescent depression, adolescent close friends who smoked, adolescent body mass index, race/ethnicity, and sex in the logistic regression model (light smoking, AOR = 2.45 [95% CI: 2.00, 3.00]; average smoking, AOR = 5.57 [95%CI: 4.17, 7.43]; and heavy smoking: AOR = 5.23 [95% CI: 3.29, 8.31]). Also significant in the adjusted analysis were having adolescent close friends who smoked; being overweight in adolescence; non-Hispanic white race; and being male.

## 4. Discussion

The purpose of this study was to examine the association between the light smoking behavioral pattern in adolescence with smoking in young adulthood. Smoking behavioral patterns in the study sample at baseline were 15.9% light smoking, 6.8% average smoking, and 3.6% heavy smoking. In the Wave IV follow-up, there were 38.4% young adults who smoked. In logistic regression analysis, adolescents with a light smoking behavioral pattern had an AOR of 2.45 (95%CI: 2.00, 3.00) to becoming young adults who smoked. The AOR for adolescent with average and heavy smoking behavioral patterns were 5.57 (95% CI: 4.17, 7.43) and 5.23 (95% CI: 3.29, 8.31), respectively. Other adolescent sociodemographic characteristics were also associated with becoming adult smokers. These included the number of adolescent close friends who were smokers, non-Hispanic white race, male sex, and overweight status. Depression symptoms with a cut point of 24 on the CES-D scale were positive (adjusted odds ratio [AOR] = 1.17) but were not statistically significant.

A similar result for depression was reported in a study by Lydon and colleagues in which a positive, but not statistically significant association for psychological distress and light cigarette smoking, occurred in young adult women (AOR = 1.17 [95% CI 0.90, 1.52]) [15].

### 4.1. Other Studies

Although there is limited literature on the health effects of light smoking [4], the results of this study are consistent with other research results:

- Researchers using data from the Raising Healthy Children project determined that 58% (*n* = 158) of adolescents who initiated smoking in 8th grade transitioned to daily smoking by 12th grade [23]. The other risk factors included adolescent depression and peers’ smoking (in single predictor models) [23]. Peers’ smoking remained a risk factor in multivariate analyses [23].
- In a longitudinal, drug abuse prevention study in Kansas City, Missouri (*n* = 1017), baseline middle school “low” tobacco users (defined as 0–4 cigarettes) were likely to be nonsmokers as adults (OR= 9.11 [95% CI 2.56, 32.46]) [17].
- In a 4-year (2002–2006) Massachusetts longitudinal school study (*n* = 970), 1.1% of children in 6th grade at baseline (mean age = 12.2 years) had used tobacco in the previous 30 days, and 38.2% developed ICD-10-defined dependence on tobacco [24].

### 4.2. Study Limitations and Strengths

The study limitations include the use of self-report data which have the potential of recall bias. The depression symptoms variable was identified as a “yes” response with a definition of 24 as the cut point on the CES-D scale; a different cut point may affect the results.

There were 1,427 of the original 6,472 (22.0%) participants who were lost-to-follow-up; however, the baseline characteristics of the participants lost-to-follow-up were similar to the baseline characteristics of the participants in the study (75.8% did not have smoking behavioral patterns, 14.6% had light smoking behavioral patterns, 6.6% had average smoking behavioral patterns, and 3.1% had heavy smoking behavioral patterns). An additional limitation is that there are various definitions for light smoking which affects the comparability across studies. The strength of this study lies in the use of a nationally representative longitudinal study as the data source and the large number of participants in the study, thus making the results more generalizable.

### 4.3. Implications for Educators/Clinicians

Adolescents have several pathways to light cigarette smoking behavior including the cost of cigarettes, peer pressures at social events, emotional distress, lack of identification as smokers, and less motivation to quit [13]. Light smoking behavioral patterns in adolescence are harmful behaviors and are predictive of future adult cigarette smoking. Consistent light smoking has substantial associated risks to health [4].

Adolescents are educated about the harm of smoking; however, many adolescents may not consider light smoking as being “smoking.” There remains a need for continued interventions and education programs for adolescents, particularly about light cigarette smoking. Interventions should include screening for any level of tobacco use [15]. The tobacco industry has marketing campaigns that are effective.

Efforts are needed to make adolescents aware of marketing techniques that are utilized to attract consumers. Efforts are also needed to increase awareness of the harms associated with light cigarette smoking in adolescence.

Some of the active smoking cessation/intervention activities appropriate for adolescents have been created by the American Lung Association. One such program is Teens Against Tobacco Use (TATU). TATU is a peer-education program in which middle and high school students are trained to teach elementary school students about the hazards of tobacco use. Researchers found that teens enjoy opportunities to positively influence children, and they are more likely to internalize the message when they do so [11].

## 5. Conclusions

Adolescents who initiate cigarette smoking, including light cigarette smoking behavioral patterns, are more likely to become young adults who smoke. There is a need for more research to prevent smoking initiation as outlined by the 2008 Clinical Guideline recommendations on Treating Tobacco Use and Dependence [7]. Further research is also needed to identify effective and specific counseling and medication interventions for adolescents engaging in light smoking behavioral patterns. Screening of adolescents concerning any tobacco use by all healthcare providers should be the norm [7].

## Figures and Tables

**Table 1:** Sample characteristics: national longitudinal study of adolescent to adult health, 1994–2008 (public use).

Total	Frequency Weighted percent	
	5,077	100.0
1Smoking (Wave I)		
No	3,817	73.7
1–5 cpd (light)	787	15.9
6–16 cpd (average)	320	6.8
17 or more cpd (heavy)	153	3.6
Smoking (Wave IV)		
No	3,248	61.7
1–5 cpd (light)	736	14.4
6–16 cpd (average)	680	14.7
17 or more cpd (heavy)	413	9.3
Race/ethnicity		
Non-Hispanic white	3,206	70.1
Non-Hispanic black	1,138	15.2
Hispanic	530	11.1
Other	203	3.6
Sex		
Female	2,746	49.6
Male	2,330	50.4
Age group in Wave I		
11–14 years	1,187	25.1
15–16 years	1,780	34.7
17 years and above	2,107	40.2
Dental visit (Wave I)		
Within the year	3,430	68.3
Within 1–2 years	979	19.3
Over 2 years	540	10.2
Never	114	2.2
2Close friends who smoke (Wave I)		
None	2,792	54.3
1 close friend smokes	1,006	20.5
2 close friends smoke	596	12.1
3 close friends smoke	609	13.1
Adequate sleep (Wave I)		
Yes	3,705	74.1
No	1,365	25.9
Exercised during the past week (Wave I)		
None	816	16.9
1–2 times	1,622	33.1
3–4 times	1,278	24.8
5 times or more	1,357	25.2
3Depression symptoms (Wave I)		
Yes	770	14.8
No	4,307	85.2
Body mass index (Wave I)		
0 to less than 25	3,866	76.5
25 to less than 30	780	15.6
30 and above	431	7.9

cpd: cigarettes per day.

1 Number of cigarettes smoked per day on the days in which the participant smoked during the previous month.

2 Participants were asked if any of their three best friends smoked.

3 A cut point score of 24 on epidemiologic studies depression scale.

**Table 2:** Bivariate relationships with adult smoking patterns, National Longitudinal Study of Adolescent to Adult Health, 1994–2008 [Public Use]

Adult smoking pattern: None			1–5 cpd		6–16 cpd		17+ cpd		*P*-value
			Light		Average		Heavy		
	N	wt	N	wt	N	wt	N	wt	
		Row %		Row %		Row %		Row %	
Sex									<.0001
Female	1878	66.0	376	14.0	348	14.1	144	5.9	
Male	1369	57.4	360	14.8	332	15.3	269	12.5	
Race/ethnicity									<.0001
Non-Hispanic White	1917	58.8	424	12.8	516	16.7	349	11.6	
Non-Hispanic Black	815	68.5	182	17.6	100	10.2	41	3.8	
Hispanic	374	69.0	93	18.0	46	9.4	17	3.6	
Other	cell suppressed		cell suppressed		cell suppressed		cell suppressed*		
Body mass index (Wave I)									0.0404
0 to less than 25	2527	62.7	558	14.6	496	14.2	285	8.5	
25 to less than 30	455	57.0	118	14.1	119	16.2	88	12.7	
30 and above	266	60.0	60	12.7	65	17.1	40	9.8	
Dental visit (Wave I)									0.1855
Within the year	2221	62.2	510	14.7	438	14.1	261	9.0	
Within 1–2 years	612	60.7	146	14.7	131	14.8	90	9.8	
Over 2 years	324	58.6	66	11.6	96	19.2	54	10.6	
Never	cell suppressed		cell suppressed		cell suppressed		cell suppressed*		
1Close friends who smoke (Wave I)									<.0001
No close friends smoke	2065	71.7	347	12.6	250	10.4	130	5.4	
1 close friend smokes	604	58.7	174	16.5	152	16.4	76	83	
2 close friends smoke	298	47.2	85	14.3	126	22.0	87	16.5	
3 close friends smoke	231	37.4	121	19.0	143	23.5	114	20.1	
Adequate sleep (Wave I)									0.8135
Yes	2372	61.7	531	14.1	503	14.9	299	9.3	
No	871	61.3	205	15.3	175	14.1	114	9.3	
Exercised during the past week (Wave I)									.1528
None	5034	60.6	112	14.1	112	13.8	89	11.5	
1–2 times	1059	62.0	219	13.5	228	16.5	116	8.0	
3–4 times	803	59.9	211	16.4	160	13.7	104	9.9	
5 times or more	879	63.5	194	13.6	180	14.0	104	8.9	
2Depression symptoms (Wave I)									<.0001
Yes	427	53.6	129	15.8	124	17.1	90	13.5	
No	2821	63.0	607	14.1	556	14.3	323	8.5	
Smoking (Wave I)									<.0001
No	2785	70.7	484	12.5	358	10.8	190	6.0	
1–5 cpd (light)	352	44.4	170	20.8	174	22.7	91	12.2	
6–16 cpd (average)	76	23.8	64	20.8	108	33.7	72	21.7	
17 or more cpd (heavy)	35	23.7	18	11.6	40	24.6	60	40.1	

N= number of participants; wt %= weighted percentage; cpd=cigarettes per day smoked on the days in which the participant smoked during the previous month

* Cells were suppressed due to cell sizes.

1 Participants were asked if any of their three best friends smoked.

2 Cut point was a score of 24 on the Center for Epidemiologic Studies Depression Scale.

**Table 3:** Logistic regressions of adolescent smoking patterns on adult smoking, National Longitudinal Study of Adolescent to Adult Health (Add Health), 1994–2008 [Public Use]

Unadjusted Odds ratio [95% confidence interval]		Adjusted Odds ratio [95% confidence interval]
Adolescent smoking		
No	reference	reference
1–5 cpd (light)	3.03 [2.50, 3.67]	2.45 [2.00, 3.00]
6–16 cpd (average)	7.72 [5.82, 10.22]	5.57 [4.17, 7.43]
17 or more cpd (heavy)	7.78 [5.09, 11.89]	5.23 [3.29, 8.31]
1Adolescent close friends who smoked		
No close friends smoke		reference
1 close friend smokes		1.37 [1.13, 1.66]
2 close friends smoke		1.56 [1.23, 1.98]
3 close friends smoke		1.81 [1.44, 2.26]
2Depression symptoms in adolescence		
No		reference
Yes		1.17 [0.95, 1.44]
Adolescent body mass index		
Normal		reference
Overweight		1.25 [1.02, 1.53]
Obese		1.07 [0.84, 1.36]
Race/ethncity		
Non-Hispanic white		reference
Non-Hispanic black		0.87 [0.63, 1.20]
Hispanic		0.75 [0.60, 0.95]
Other		0.86 [0.56, 1.32]
Sex		
Female		reference
Male		1.51 [1.32, 1.73]

cpd=cigarettes per day smoked on the days in which the participant smoked during the previous month

1 Participants were asked if any of their three best friends smoked.

2 Cut point was a score of 24 on the Center for Epidemiologic Studies Depression Scale.

Wald test p<.0001 for both unadjusted analysis and adjusted model.

Adjusted model includes depressive symptoms in adolescence, adolescent close friends who smoked, adolescent body mass index, race/ethnicity and sex.
